# Porous Silicon on Paper: A Platform for Quantitative Rapid Diagnostic Tests

**DOI:** 10.1021/acsami.4c18940

**Published:** 2025-01-15

**Authors:** Huijin An, Simon J. Ward, Rabeb Layouni, Paul E. Laibinis, Andrea K. Locke, Sharon M. Weiss

**Affiliations:** † Interdisciplinary Material Science Program, Vanderbilt University, Nashville, Tennessee 37235, United States; ‡ Department of Electrical and Computer Engineering, Vanderbilt University, Nashville, Tennessee 37235, United States; § Department of Chemical and Biomolecular Engineering, Vanderbilt University, Nashville, Tennessee 37235, United States; ∥ Department of Chemistry, Vanderbilt University, Nashville, Tennessee 37235, United States; ⊥ Department of Biomedical Engineering, Vanderbilt University, Nashville, Tennessee 37235, United States

**Keywords:** porous silicon, optical biosensor, paper-based sensor, rapid diagnostic test, membrane, label-free

## Abstract

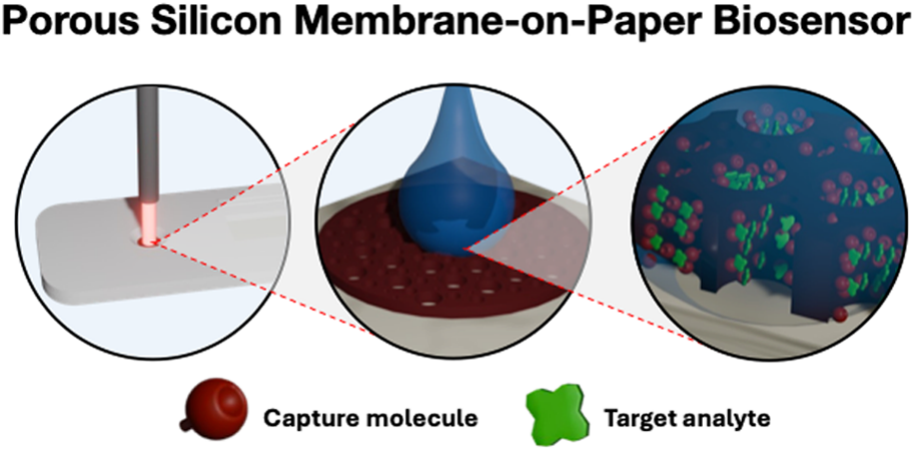

Porous silicon (PSi) thin films on silicon substrates have been extensively investigated in the context of biosensing applications, particularly for achieving label-free optical detection of a wide range of analytes. However, mass transport challenges have made it difficult for these biosensors to achieve rapid response times and low detection limits. In this work, we introduce an approach for improving the efficiency of molecule transport in PSi by using open-ended PSi membranes atop paper substrates in a flow-through sensor scheme. The paper substrate provides structural support as well as an efficient means of draining solutions from the PSi membrane without the use of an external pump and microfluidic channels. Distinct changes in the reflectance properties of the PSi membrane are measured when molecules are captured in the membrane. A concentration-dependent response of the sensor for protein detection is demonstrated. Factors influencing the interaction time of molecules in the PSi membrane and the drying time of the membrane, which directly affect the detection sensitivity and overall testing time, are discussed. The demonstrated performance of the PSi-on-paper sensor establishes the feasibility of a platform for low-cost rapid diagnostic tests with a highly sensitive, quantitative readout.

## Introduction

Rapid diagnostic tests (RDTs) are indispensable in healthcare, providing quick and accessible results for various medical conditions, including influenza, malaria, and COVID-19.^1−3^ While paper-based immunoassays are among the most common RDTs due to their affordability and user-friendly nature, challenges persist, particularly regarding accurate quantification of color changes and the ability to detect low concentrations of analytes.^1,4−8^ To enable quantification, external devices such as scanners, phone cameras, and portable spectrometers have been used when signal changes are sufficiently distinct over the relevant target molecule concentration range.^9−11^ To increase the detection sensitivity as well as provide improved signal quantification, chemiluminescent and fluorescent labels have been used, but incorporating such labels into paper-based immunoassays can increase the cost of the assay and requires a more sophisticated measurement method.^1,5,12,13^ We propose a new approach for achieving a quantifiable color change upon target molecule capture in a paper-based immunoassay based on the integration of porous silicon (PSi) free-standing membranes (FSMs) with paper-based substrates and suggest this new material combination offers a potential path forward to achieving quantitative and sensitive label-free RDTs.

PSi has been demonstrated as a promising material for biosensing, offering a large surface area for molecular capture, straightforward surface modification, easy optical readout, cost-effectiveness, and biocompatibility.^14^ Traditional PSi biosensors that utilize one or more PSi films on a silicon substrate have been demonstrated for detecting various analytes including proteins, DNA, and other small molecules in both buffered solutions and complex media.^15^ Most commonly, PSi biosensors are measured optically using a benchtop spectrometer and signal processing, but readout using a smartphone has also been reported.^16,17^ Despite their many advantages, PSi optical biosensors have not seen widespread usage, likely in part due to diffusion challenges that make it more difficult for molecules to infiltrate closed-ended nanoscale pores, which lead to reduced sensor signal changes and slower sensor response times.^18^ PSi optical biosensors typically have response times of 1–2 h.^17−20^ While it has been shown that open-ended PSi membranes can facilitate faster molecular transport and binding in the pores, prior work reporting on a PSi membrane-based optical biosensor required lithographic patterning, microfluidic channels, and an external pump.^21^

In this work, we show that adding a paper-based fluidic substrate beneath an easy-to-fabricate PSi FSM provides the necessary capillary forces to move solutions through nanoscale pores without the use of an external pump and microfluidic channels, opening the door to the realization of a paper-based PSi RDT with quantitative smartphone readout for low-cost point-of-care applications. Herein, concentration-dependent detection of streptavidin protein using the PSi-on-paper platform is demonstrated with improved molecule transport efficiency compared with similar PSi films that remain on the silicon substrate.

## Experimental Procedures

### Materials

Single-side polished boron-doped p-type silicon wafers (⟨100⟩, 0.01–0.02 W-cm, 500–550 μm) were purchased from Pure Wafer, WRS Materials Company. Hydrofluoric acid (HF, 48–51% solution in DI water), 3-aminopropyltriethoxysilane (APTES, 99%), EZ-Link sulfo-NHS-biotin, streptavidin, methanol, ethanol (200 proof), phosphate-buffered saline (PBS), sodium hydroxide (NaOH), and Whatman filter paper grade 4 were purchased from Thermo Fisher Scientific. Mini Trans-Blot filter paper (henceforth termed the absorbent pad) was purchased from Bio-Rad. Deionized (DI) water with a resistivity of 15 MW-cm was obtained from a Millipore Elix water purification system. In all cases, water refers to DI water in this work.

### Porous Silicon Free-Standing Membrane (PSi FSM) Fabrication

PSi FSMs (∼2 cm^2^) were fabricated by electrochemical etching of p-type silicon at room temperature in an electrolyte solution containing a 3:7 volume ratio of HF and ethanol. First, a sacrificial layer of PSi was etched at a current density of 80 mA/cm^2^ for 50 s and was subsequently removed in a 1 M solution of NaOH. After rinsing the silicon with water and ethanol to remove residual NaOH, the following current density sequence was applied to create a symmetric 3-layer PSi structure: (1) 80 mA/cm^2^ for 42 s, (2) 40 mA/cm^2^ for 846 s, (3) 80 mA/cm^2^ for 42 s. Higher applied current densities lead to higher porosity PSi with larger pores, and longer etching times lead to thicker PSi layers. To detach the 3-layer PSi structure from the silicon substrate, a liftoff process that electropolished the surface was carried out by applying a high current density of 500 mA/cm^2^ for 1.7 s, repeated twice. The resulting free-standing PSi membrane was then washed with ethanol and transferred onto Whatman paper to dry in air at room temperature. The symmetric design of the PSi FSM was selected to minimize the stresses formed during subsequent thermal oxidation and drying steps after other surface treatments.

### PSi FSM Surface Functionalization

The as-anodized PSi FSM underwent thermal oxidation in air at a temperature of 500 °C for a duration of 1 h. This thermal treatment was carefully executed to mitigate thermal shock, involving a gradual temperature ascent for 100 min to the desired level, followed by a slow descent for 60 min to room temperature. Subsequently, the thermally oxidized PSi FSM was immersed in a 4% solution of APTES for 10 min in a glass Petri dish, followed by a 15 min soak in methanol to eliminate any unreacted APTES. After being rinsed, it was dried on Whatman paper, and then the APTES-modified PSi FSM was annealed at 150 °C in an oven in air for 15 min. Following the annealing step, the PSi FSM was submerged in a solution of 0.01 mg/mL of sulfo-NHS-biotin in PBS in a Petri dish and incubated overnight. Finally, the biotin-conjugated PSi FSM was subjected to a 1 h soak in DI water and ethanol to remove any residual biotin and was subsequently dried on Whatman paper.

### PSi FSM on Paper Assembly

The biotin-conjugated PSi FSM was placed on top of a fresh piece of Whatman paper as the first step in the assembly of the sensor. Next, to facilitate the withdrawal of solutions from the PSi FSM, an absorbent pad was positioned beneath the Whatman paper. A hydrophobic, wax-printed Whatman paper featuring a 3 mm aperture was placed on top of the PSi FSM to provide a reference point for sensor measurements and to limit the area into which solutions could infiltrate the PSi FSM. A custom plastic cartridge featuring a flat hole (i.e., not funnel-shaped) that was aligned with the aperture in the wax-printed Whatman paper was utilized for the assembly of the PSi FSM on a paper sensor. Six screws along the outer perimeter of the cartridge secured the assembly. This design reduced intermembrane spacing and alleviated inhomogeneous pressure that is common near the funnel-shaped hole in commercialized cartridges.

### Streptavidin Sensing

The high binding affinity biotin–streptavidin assay was used as a model system to demonstrate specific molecular detection using the new PSi on a paper sensor platform. Streptavidin (SA) was dissolved in water to obtain the desired concentration. The PSi-on-paper sensor platform was exposed to streptavidin target molecules of concentrations between 0 and 30 μM. First, to ensure proper operation of the sensor, 10 μL of water was pipetted on the PSi FSM through the small hole in the plastic cartridge. Next, 20 μL of streptavidin of a given concentration (0–30 μM) was introduced to the sensor, followed by 10 μL of water to remove any unbound molecules. For the initial streptavidin sensing experiment, a second 10 μL of water was introduced to the sensor to determine whether additional unbound SA molecules remained after the first water rinse. This second water rinse was not needed in subsequent experiments. For comparison, traditional on-substrate (i.e., on a silicon substrate) PSi sensor experiments were conducted with a single-layer PSi film fabricated with the same conditions as the sensing layer of the PSi FSM (i.e., a sacrificial layer followed by application of 80 mA/cm^2^ for 42 s). In these experiments, the on-substrate PSi samples were cleaved to a size of 4 mm by 4 mm and received the same surface functionalization as the PSi FSM samples, including oxidation, APTES attachment, and biotin modification. The on-substrate PSi samples were incubated with 20 μL of streptavidin over the same 0–30 μM concentration range for different time durations up to 2 h. After each incubation step, the on-substrate PSi samples were rinsed with water in a Petri dish on a shaker table for 5 min and then dried with nitrogen gas. For each streptavidin concentration, three individual PSi-on-paper sensors and three individual on-substrate PSi samples were tested to establish the reproducibility and repeatability.

### Optical Reflectivity Measurements

Real-time reflectance measurements were carried out during the sensing experiments, similar to those reported previously.^22^ A white light source (Oriel 6000 Q Series quartz tungsten halogen lamp), an Ocean Optics reflectance probe, and a fiber-coupled Ocean Optics USB4000 CCD spectrometer were used to collect reflectance spectra over a spot size of approximately 3 mm in diameter at the center of the PSi FSM sensors and on-substrate PSi samples. Reflectivity data were recorded every 20 s until the end of the measurement, starting at least 2 min before the introduction of the first droplet to each sensor, with a spectral acquisition time of 10 ms over a wavelength range of 500 to 1000 nm. We note that a fan was employed during all optical measurements of the PSi-on-paper sensors to reduce the drying time of the PSi-on-paper tests, as discussed in the Results and Discussion section.

## Results and Discussion

### Characterization of PSi Free-Standing Membrane

**Figure 1:**
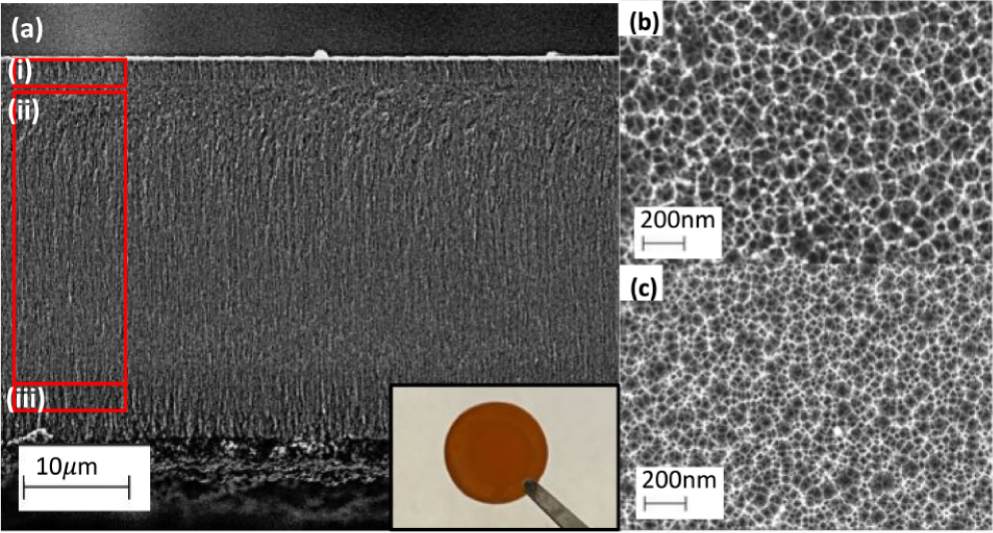
Images of the PSi FSM. (a) Cross-sectional SEM image of the edge of the membrane region with the three PSi layers labeled as i, ii, and iii. Inset shows photograph of PSi FSM held by tweezers. Top-view SEM images of (b) PSi sensing layer and (c) underlying PSi structural support layer.

Representative PSi samples were fabricated for characterization by scanning electron microscopy (SEM, Figure 1). Top-view SEM images were taken using samples that remained on the silicon substrate, and cross-sectional SEM images were taken using a complete three-layer FSM. We note that two separate on-substrate samples were fabricated to enable top-view SEM imaging of PSi films with the same properties as the top and middle PSi layers of the FSM. For each of these on-substrate samples, a sacrificial layer was first etched and removed before etching a PSi film with the same current density and etching time as the corresponding layer in the FSM; the sacrificial layers were etched with the same current density used to form the subsequent PSi film. SEM analysis of top-view images (Figure 1b,c) reveals distinctive pore size distributions within the top and middle layers, as expected.

The top layer fabricated at a higher current density has a larger average pore diameter of 47 nm, while the middle layer has an average pore diameter of 34 nm.^23^ Details about the SEM image analysis to determine average pore diameter are discussed in the Supporting Information; pore size distributions in each layer are shown in Figure S1 and quantified in Table S1. The cross-sectional SEM image (Figure 1a) shows the three-layer structure of the PSi FSM, along with an additional layer at the bottom that was created during the liftoff process. From this cross-sectional image, we determined that the top and middle layers of the PSi FSM had thicknesses of 1.2 and 25 μm, respectively. The third layer, directly below the middle layer, had the same thickness as the top layer. Top-view SEM images of the bottom of the PSi FSM are shown in Figure S2, revealing substantially larger pores, which are likely due to the electropolishing step used to detach the PSi from the silicon substrate.

The three-layer symmetric structure of the PSi membrane was designed to facilitate the mass transport of molecules while ensuring structural robustness. The larger pore size and smaller thickness of the first PSi layer allow easy access of molecules to this sensing layer. We term this layer the sensing layer, because analysis of the optical reflectance measurements focuses on optical thickness changes that occur in this layer alone, as discussed below. Although hindered diffusion has been reported in PSi on-substrate, larger pore sizes and thinner films are known to improve molecular infiltration and binding efficiency.^18^ Moreover, the open-ended pores in the FSM further improve mass transport of molecules compared to on-substrate closed-ended PSi films as the solution is pulled through the membrane and exits on the opposite side of the membrane into the Whatman paper and absorbent pad. The second layer of the PSi FSM is designed to have smaller pores and a larger thickness to improve the structural robustness of the membrane. The overall thickness of the membrane was targeted to be at least 20 μm to enable relatively easy handling during the fabrication and fluidic chip assembly. The pore size of all layers could be modified, as necessary, for example, if pore clogging becomes an issue with the detection of larger species. However, there must be a refractive index contrast, which manifests as a porosity and pore size contrast between the top and middle layers to maintain the benefits of a thinner sensing layer that can be monitored optically. The third layer of the PSi FSM was designed to form a symmetric structure, minimizing stress during the thermal oxidation process. Empirically, we found that two-layer membranes were much more likely to curl after oxidation and after subsequent functionalization steps that involved wetting and then drying the PSi FSM.

**Figure 2:**
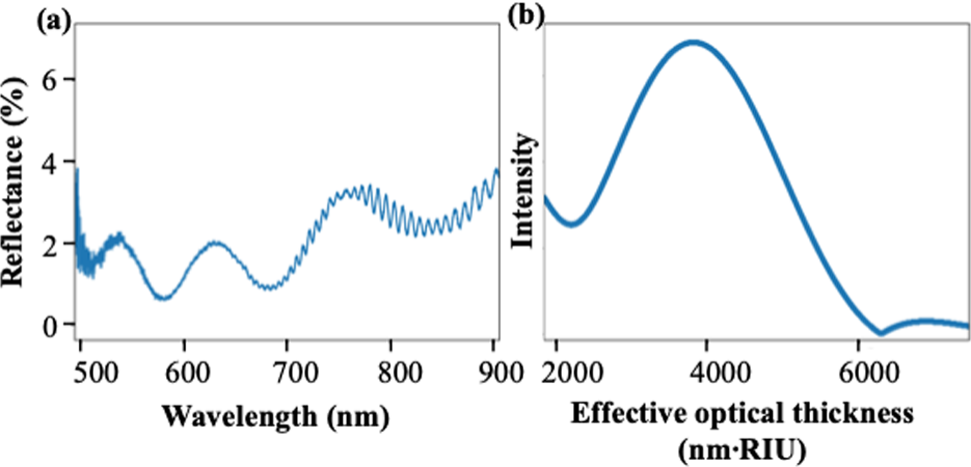
(a) Reflectance spectrum of multilayer PSi FSM. (b) Effective optical thickness (EOT) of top sensing layer of the multilayer PSi FSM.

As shown in Figure 2a, the reflectance spectrum of the three-layer PSi FSM is characterized by interference fringes. In order to accurately monitor solution infiltration and molecular binding in the different layers of the PSi FSM, we use reflective interferometric Fourier transform spectroscopy (RIFTS).^16,24^ The RIFTS technique utilizes a fast Fourier transform (FFT) to isolate the different frequency components of the reflectance spectrum that originate from interference of light in the different layers of the three-layer FSM. Peaks in the FFT spectrum correspond to the effective optical thickness (EOT) of each individual layer and the different combinations of layers of a multilayer thin film where EOT = 2 *nL*, where *n* is the effective refractive index of a particular PSi layer or combination of layers and *L* is the corresponding thickness.^25^ We note, however, that because the middle PSi layer of the FSM is not a thin film (i.e., thickness is much greater than the wavelength of light), in addition to the absorption of visible light that occurs in PSi, we can only clearly resolve the FFT peak corresponding to interference between light reflecting off the top and bottom surfaces of the top sensing PSi layer of the FSM (Figure 2b).

To verify that the FFT peak at an EOT near 4000 nm·RIU (refractive index unit) corresponds to the top sensing layer of the PSi FSM, optical reflectance measurements and RIFTS analysis of a single-layer PSi on-substrate sample formed with the same electrochemical etching conditions as the top sensing layer of the membrane were carried out. As shown in Figure S3, the EOT of this single-layer on-substrate sample is near 4000 nm·RIU, confirming that the peak shown in Figure 2b corresponds to the EOT of the sensing layer of the PSi FSM.

The EOT of the sensing layer of the PSi FSM changes when the solution infiltrates the top layer and/or molecules bind in the top layer. Because solution filling the pores causes a larger effective refractive index change (i.e., when all air in the pores is replaced by the solution) than the binding of molecules on the pore walls, the EOT increase due to solution infiltration is much larger than the EOT increase due to molecular binding. In this way, the magnitude of the EOT change can provide insights into the physical changes occurring inside the pores of the top sensing layer. Moreover, these insights can be made on the top sensing PSi layer, independent of what is happening inside the supporting PSi layers.

### Surface Modification and Sensor Assembly

**Figure 3:**
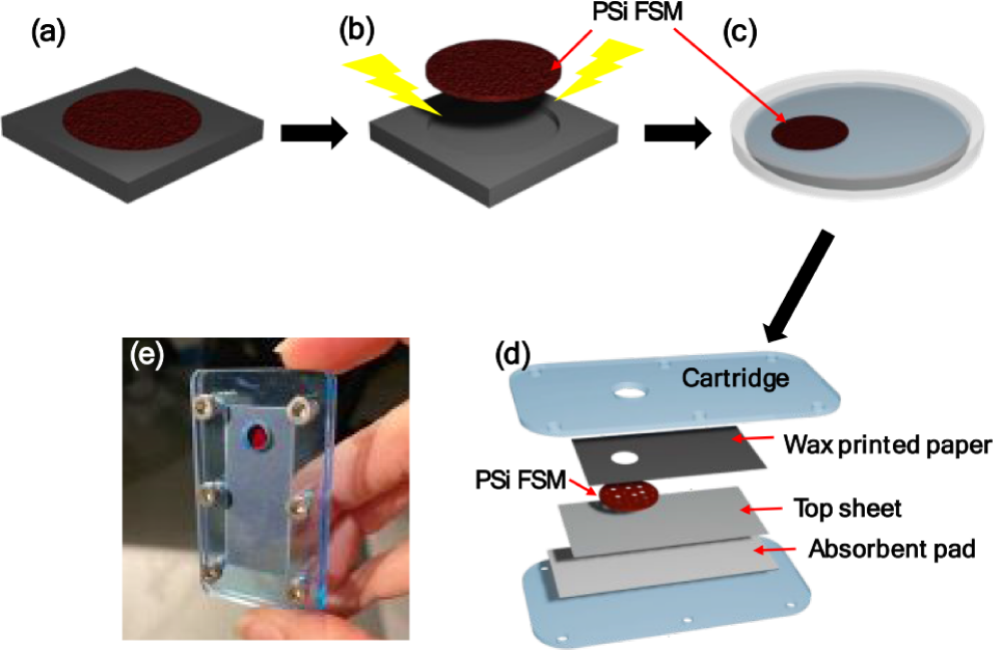
Schematic of PSi-on-paper fabrication process. (a) PSi three-layer film formation by electrochemical etching; (b) liftoff process to remove three-layer PSi FSM from silicon substrate; (c) surface modification of PSi FSM in Petri dish carried out after thermal oxidation; (d) PSi-on-paper sensor assembly with wax-printed paper, PSi FSM, Whatman paper top sheet, and absorbent pad within a plastic cartridge. (e) Photo of PSi-on-paper sensor where red circle is PSi FSM visible through aperture in wax-printed paper.

After fabrication of the PSi FSM, thermal oxidation, and surface modification with APTES and biotin, the PSi FSM-on-paper sensor was assembled in a plastic cartridge. Figure 3 shows the various steps in this process.

We note that thermal oxidation was chosen as the method to passivate the hydrogen-terminated surfaces of the as-anodized PSi FSM, which are hydrophobic and prone to corrosion in aqueous solutions,^22^ due to the simplicity of the process, although other methods could be utilized.^26,27^ A relatively low temperature of 500 °C was selected to minimize thermal stresses while still providing the necessary oxide surface for subsequent surface modification with APTES.^28^ Despite the relatively low oxidation temperature and the slow ramp-up and cool-down procedures utilized, some PSi FSMs curled slightly after oxidation. These FSMs were still usable as long as a flat region was probed for reflectance measurements. A key aspect of the assembly of the biotin-modified PSi FSM and paper substrate layers is ensuring intimate contact between these layers to prevent leakage and promote fluid transport between the layers. Accordingly, the plastic cartridge was designed to minimize the gap between the PSi FSM, Whatman paper, and absorbent pad by applying a uniform pressure across all layers.

### PSi-on-Paper Sensing

**Figure 4:**
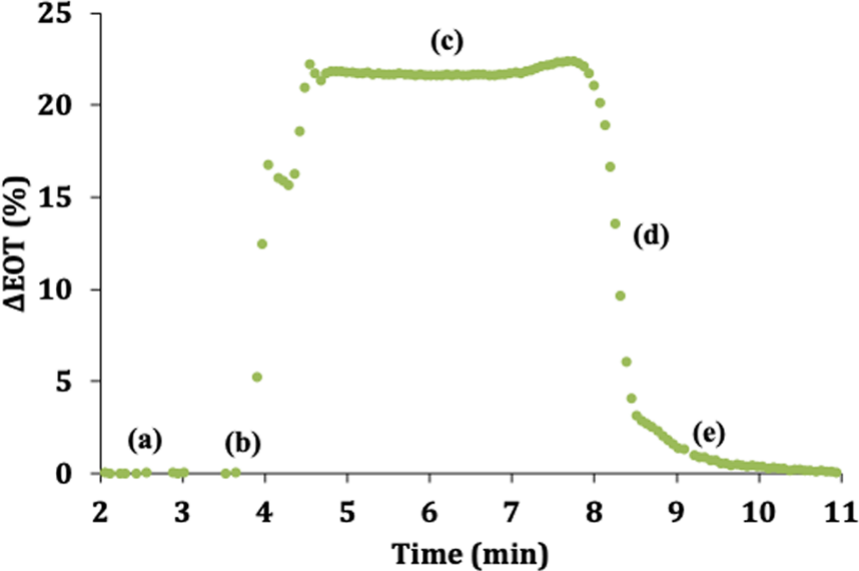
Real-time change in EOT of sensing layer of PSi-on-paper sensor upon exposure to 10 μL droplet of water: (a) initial baseline EOT when PSi FSM is dry, (b) introduction of water droplet resulting in filling of pores with water, (c) pores are completely full of water (i.e., “contact time”), (d) draining stage when water drains from the pores and is absorbed into the absorbent pad, and (e) return to the fully dry state of PSi FSM.

Real-time reflectance measurements enable the monitoring of fluid transport through the PSi FSM and quantification of molecular binding in the top sensing layer. The time-dependent EOT changes of the top sensing layer are calculated by applying the RIFTS method to the real-time reflectance data. To understand the evolution of the EOT change when a solution passes through the PSi FSM, we first acquired a baseline measurement of the dry PSi-on-paper sensor (Figure 4a) then introduced 10 μL of water into the PSi-on-paper system. The introduction of the water droplet resulted in an increase in the EOT as water filled the pores of the top sensing layer of the PSi FSM (Figure 4b). After ∼1 min, the EOT change saturated when the pores of the sensing layer were completely filled with water (Figure 4c). The pores in the sensing layer remain filled with water for ∼6 min as water both entered and exited the pores (i.e., “contact time”), and then, the EOT rapidly decreased as the water drained from the pores in the sensing layer and was absorbed into the absorbent pad (Figure 4d). This rapid decrease in EOT was followed by a more gradual decrease and return to the baseline EOT value (at ∼10 min), corresponding to that of the dry PSi FSM (Figure 4e).

We attribute the initial faster decrease in EOT to when the water droplet is draining from the PSi FSM and being absorbed by the Whatman paper and absorbent pad below the PSi FSM. We believe the final slower change in EOT that more gradually approaches the baseline value can be explained by the wet paper and pad holding water below the PSi FSM, which provides a humid environment with the possibility of having water reabsorbed by the PSi FSM. We verified this assertion by carrying out a control experiment in which 10 μL of water was introduced directly into the absorbent pad. As shown in Figure S4, water was indeed wicked up from the absorbent pad through the Whatman paper into the pores of the sensing layer of the PSi FSM. Hence, in future work, the design of the PSi on paper sensor will be modified, for example, by using an absorbent pad that can absorb larger amounts of water more efficiently, to enable improved transport of liquid away from the PSi FSM, which will prevent reabsorption and allow the PSi FSM to dry more quickly. As stated in the Materials section, a fan was employed during subsequent optical measurements reported here to reduce the time corresponding to part (e) in Figure 4 because the prolonged drying time is not related to the intrinsic draining of the PSi FSM.

Next, biotin-functionalized PSi-on-paper sensors were employed for the quantitative detection of the SA protein. We note that each sensor was used in only one experiment and then was discarded, which is consistent with RDT usage. In our first experiment with SA, we verify that a permanent EOT change results from the capture of SA in the pores, and we determine how many rinsing steps are needed to remove unbound SA from the pores. In our subsequent experiments, we demonstrate a concentration-dependent change in the EOT for SA detection and establish a more efficient protocol for analyte introduction and rinsing.

**Figure 5:**
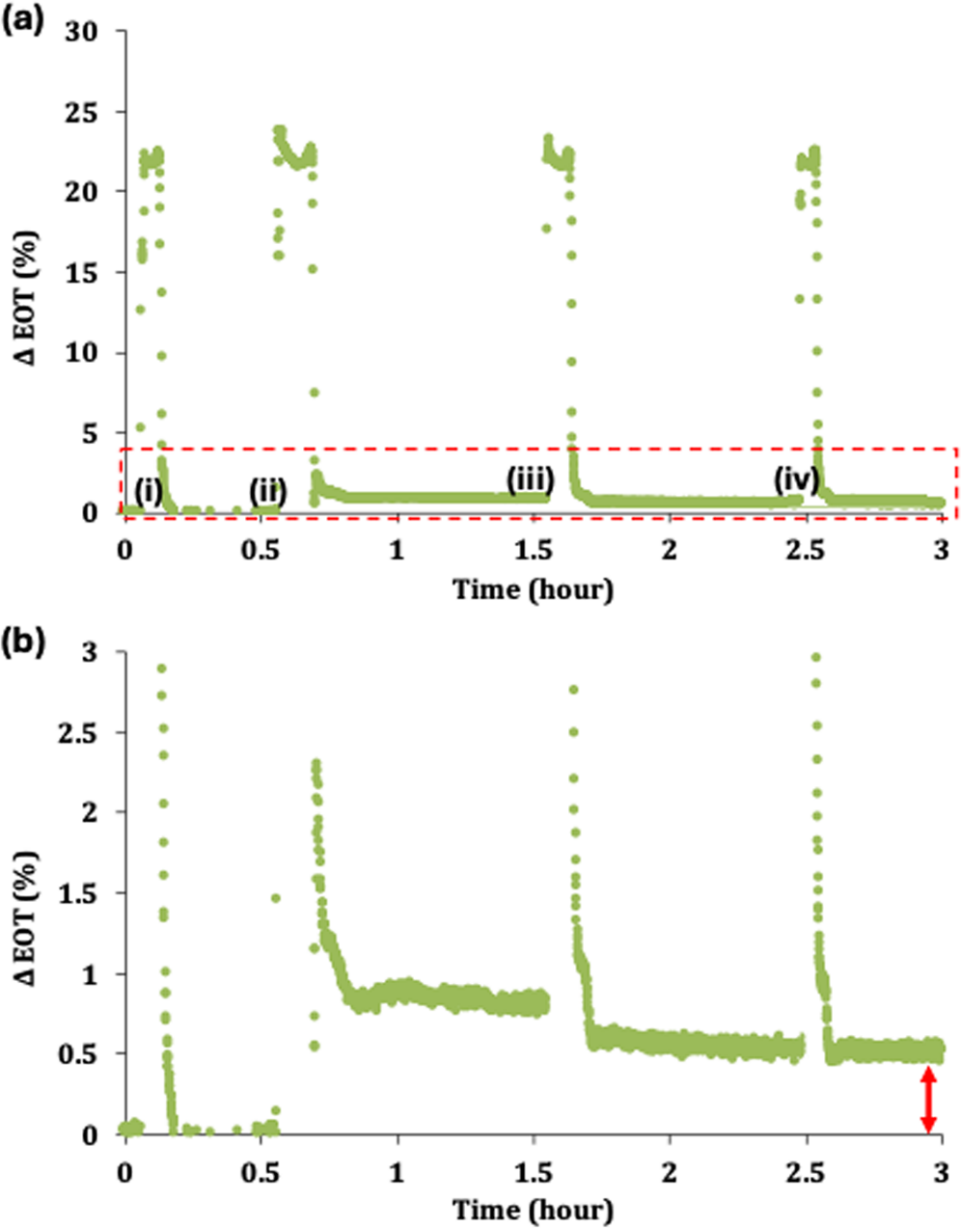
Real-time PSi-on-paper sensor response to 20 μL of 10 μM SA solution. (a) Long-duration experiment in which sensor was dried between steps of (i) initial 10 μL water droplet, (ii) 20 μL of 10 μM SA solution, and (iii, iv) two 10 μL water rinsing steps. (b) Zoomed-in view of EOT changes in long-duration experiment shown in (a).

Figure 5 shows the results of our first SA sensing experiment. We started by verifying the operation of the PSi-on-paper sensor through the introduction of a 10 μL water droplet, similar to what is shown in Figure 4. As expected, the EOT returned to the baseline value after the water drained from the pores, and the PSi FSM returned to the dry state. Then, the PSi-on-paper sensor was exposed to 20 μL of 10 μM SA solution. A rapid increase in the EOT occurred as the SA solution filled the pores. We note that the contact time when the SA solution completely filled the pores and SA molecules were captured by surface-attached biotin molecules was longer than that for the water droplet primarily because the SA solution volume was larger. Similar to what was observed when the water droplet was introduced to the sensor, the contact time was followed by an early rapid decrease in EOT and then a more gradual decrease in EOT.

However, after exposure to SA, the EOT does not return to the baseline value because SA molecules remain in the pores of the sensing layer, attached to biotin, after the solution drains from the pores. The EOT change after SA exposure was approximately 0.8%. Next, a water rinsing step was carried out that introduced 10 μL of water to the PSi-on-paper sensor to remove any unbound SA from the PSi FSM. This step is crucial for ensuring that the sensor’s response is specific to the captured SA molecules. After this rinsing step, the EOT change compared to the initial baseline was approximately 0.5%, indicating that there was unbound SA in the pores. To ensure all unbound analytes were removed, a second 10 μL water rinse was conducted.

No additional change in EOT was observed after the second water rinse, suggesting that one water rinse is sufficient to remove the unbound analyte. A control experiment was carried out in which the PSi FSM was modified with APTES but no biotin and then exposed to SA. As shown in Figure S5, there is less than a 0.1% change in EOT due to nonspecific binding of SA in the pores, verifying that the majority of the signal change in Figure 5 is due to specific capture of SA by incorporated biotin molecules. The overall time duration of this first SA sensing experiment was longer than necessary for two reasons. First, we carried out an extra water rinse step, and second, as mentioned earlier, the current configuration of the PSi-on-paper test introduces an extended drying time due to the sensitivity of the PSi FSM to a wet underlying absorbent pad (see Figure S4).

Complete drying of the absorbent pad was necessary to achieve a stable signal after the solution was drained from the PSi FSM. We note that slight variations in the EOT in the relatively stable signal regions may be attributed to slight changes in moisture levels in the absorbent pad. Furthermore, we note that the ability of the absorbent pad to continue to absorb liquid after an initial wetting becomes reduced, which further extended the drying times during this test. To reduce the overall testing duration, we next carried out another experiment using 20 μL of 10 μM SA solution, but this time added the 10 μL water droplet to rinse unbound SA molecules in the pores shortly after visually observing the disappearance of the droplet from the top layer of the PSi FSM.

**Figure 6:**
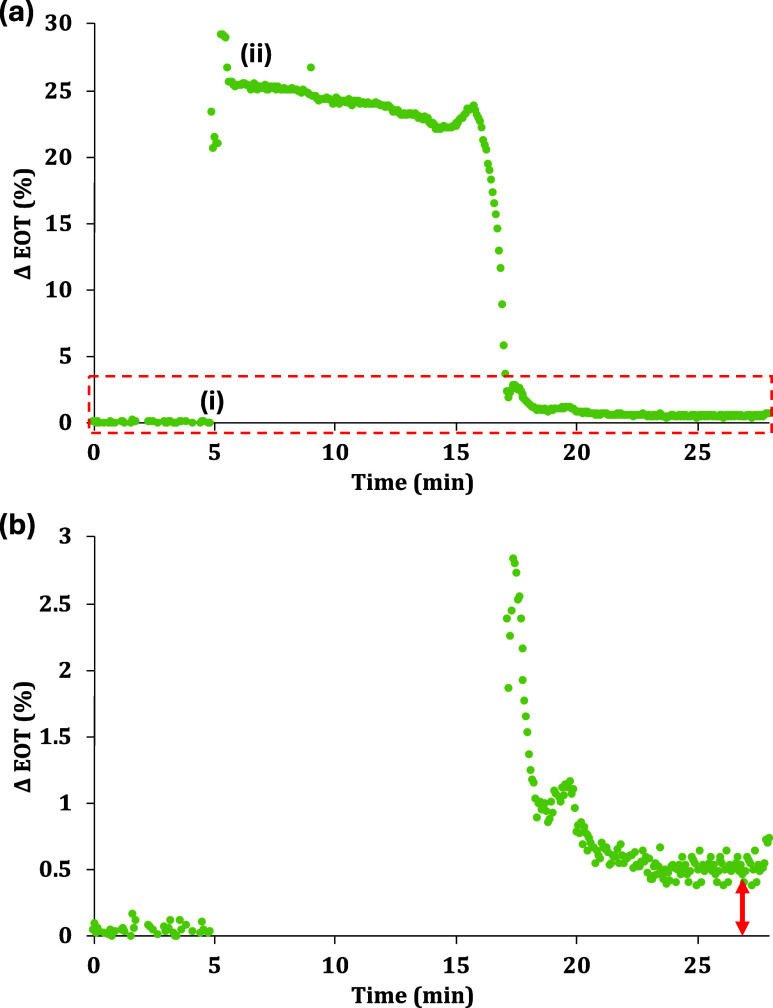
Modified real-time PSi-on-paper sensor experiment for the detection of 20 μL of 10 μM SA solution. (a) Short duration experiment in which only one 10 μL water-rinsing step was performed shortly after the SA droplet disappeared from view at the top of the PSi FSM: (i) introduction of 20 μL of 10 μM SA solution, (ii) 10 μL water droplet introduced to rinse out unbound SA. (b) Zoomed-in view of EOT changes in short duration experiment shown in (a).

As shown in Figure 6a, adding two 10 μL droplets within a short time frame led to minor variability in the EOT signal before the signal stabilized but afterward followed the same trend as shown in the earlier experiment (Figure 5). With a time duration of approximately 30 min, this PSi-on-paper sensor test showed similar ΔEOT results as before and further establishes the feasibility of the PSi-on-paper sensor platform for rapid diagnostic tests. We note that the PSi-on-paper sensor platform can be adapted to detect the presence of a wide variety of biomarkers by modifying the PSi FSM with the appropriate antibody or another type of capture agent.

**Figure 7:**
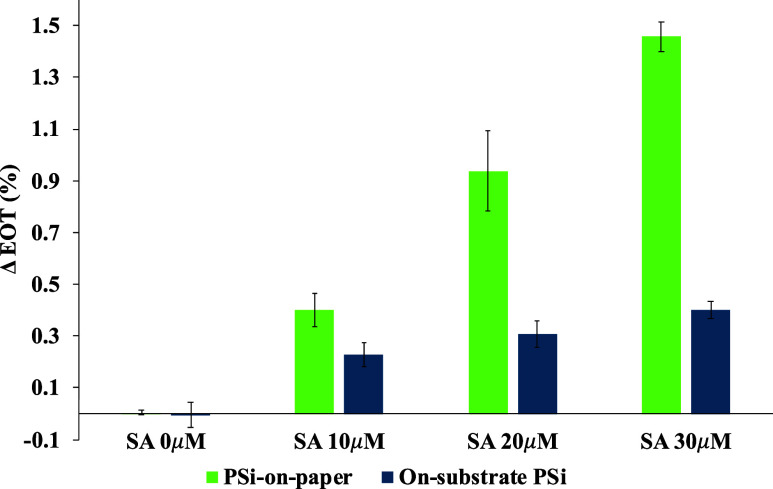
Effective optical thickness changes of PSi-on-paper and on-substrate PSi sensors exposed to different concentrations of SA solution for a similar contact time of approximately 6 min. Error bars represent the standard deviation of three separate experiments.

Finally, PSi-on-paper sensors were exposed to different concentrations of SA to demonstrate the quantitative sensing capabilities of the PSi-on-paper platform. In addition, to benchmark the concentration-dependent PSi-on-paper sensor response, we also exposed on-substrate PSi sensors to the same concentrations of SA for the same contact time of 6 min. For these experiments, three individual samples of each type of PSi sensor were tested in triplicate to demonstrate repeatability and reproducibility. Figure 7 illustrates the changes in EOT of the PSi on-paper and on-substrate sensors.

For every SA concentration, the PSi-on-paper sensors exhibit a larger change in EOT. This result is expected as the open-ended PSi FSMs enable more efficient mass transport of molecules compared with the on-substrate PSi sensors with closed-ended pores. Importantly, unlike prior sensing experiments with PSi membranes,^21^ the PSi FSMs in the paper-based sensor platform required no syringe pump or microfluidics to realize the improved mass transport and shorter response time for a given optical signal change. The response for both the PSi-on-paper and on-substrate PSi sensors was linear with SA concentration (R^2^ > 0.92), with the PSi-on-paper sensor platform exhibiting a 3.7 times higher detection sensitivity.

We note that when the on-substrate PSi sensors were incubated in SA solutions for longer time durations, larger EOT changes were measured (see Figure S6 and Supporting Information), which suggests that larger optical signal changes after exposure to a given SA concentration can also be achieved with the PSi-on-paper sensors by adjusting the incubation time.

## Conclusions

A paper-based optical biosensor utilizing an open-ended PSi FSM to enable quantitative molecular detection was designed, fabricated, and characterized. The open-ended PSi FSM facilitated more efficient transport of molecules to the active sensing region compared to PSi sensors on silicon substrates, leading to a nearly 4-fold improvement in the detection sensitivity of the streptavidin protein. A quantitative, concentration-dependent response of the PSi-on-paper sensor was demonstrated for the detection of streptavidin protein within 30 min. This response time is at least 2× faster than that for on-substrate PSi sensors. The paper-based substrates, which were utilized for the first time beneath PSi membranes, served the dual purpose of facilitating fluid drainage from the pores and supporting the PSi FSM without the need for an external pump or microfluidic channels. Importantly, the PSi FSM was designed using a 3-layer scheme that allowed (1) efficient transport and binding of target analyte within a thin top sensing layer and (2) mechanical robustness by incorporating a thicker middle layer and a thin lower layer identical to the sensing layer to create a symmetric structure that minimizes internal stresses during thermal oxidation and various drying steps during sensor preparation. This work, together with prior work demonstrating smartphone readout of PSi biosensors, establishes the feasibility of the PSi-on-paper sensor as a promising platform on which to develop quantitative rapid diagnostic tests.

## Supporting Information Available

Summary of pore size distribution of 3-layer PSi FSM; SEM images of the electropolished layer on the bottom of PSi FSM; reflectance and EOT of on-substrate PSi single-layer film; direct wetting of the absorbent pad in the PSi-on-paper sensor; nonspecific binding of streptavidin; and streptavidin sensing on-substrate with different incubation times and concentrations (PDF)
