# Supplementary material for: Porous Silicon on Paper: A Platform for Quantitative Rapid Diagnostic Tests
Source: ACS Appl Mater Interfaces. 2025 Jan 15;17(4):6024–30. doi: 10.1021/acsami.4c18940 (PMC11788981; doi:10.1021/acsami.4c18940)
Supplement: Supplementary file 1 — am4c18940_si_001.pdf [file am4c18940_si_001.pdf]

Supporting Information

POROUS SILICON ON PAPER: A PLATFORM FOR QUANTITATIVE RAPID DIAGNOSTIC TESTS

Huijin An[1], Simon J. Ward[2], Rabeb Layouni[3], Paul E. Laibinis[1,3], Andrea K. Locke[1,4,5], and Sharon M. Weiss[1,2,]*

[1] Interdisciplinary Material Science Program, Vanderbilt University, Nashville, TN 37235, USA

[2] Department of Electrical and Computer Engineering, Vanderbilt University, Nashville, TN 37235, USA

[3] Department of Chemical and Biomolecular Engineering, Vanderbilt University, Nashville, TN 37235, USA

[4] Department of Chemistry, Vanderbilt University, Nashville, TN 37235, USA

[5] Department of Biomedical Engineering, Vanderbilt University, Nashville, TN 37235, USA

Corresponding Author:

*Email: sharon.weiss@vanderbilt.edu

Table of Contents

1. Pore size distribution of 3-layer porous silicon (PSi) free standing membrane (FSM)……………………………………………………..……………………S-1
2. Electropolished layer on bottom of PSi membrane……………………………………..S-5
3. Reflectance spectrum and effective optical thickness of single-layer on-substrate PSi film……………………………………………………………………………S-6
4. Direct wetting of absorbent pad in PSi on paper sensor ………………..……………...S-7
5. Non-specific binding ……………………………………………………………...S-8
6. Streptavidin sensing in on-substrate PSi films…………………………………………S-9

1. Pore size distribution of 3-layer porous silicon (PSi) free-standing membrane (FSM)

Pore size distributions were determined from the SEM images in Figure 1b and 1c in the main text, as shown in Figure S1a. To extract the pore distribution and average pore size, analysis was carried out in MATLAB (R2022b).[1,2] Taking into account all pores in the images, and using a grayscale threshold of 100, we determined that the average pore diameter of the top sensing layer of the PSi-on-paper sensor was 47 nm, and that of the middle support layer was 34 nm. Figure S1b shows the SEM image of the top sensing layer, while Figure S1c illustrates the segmentation results of this image where the pores are highlighted in green. This grayscale threshold serves as a critical parameter in MATLAB-based image analysis for determining pore size. However, the selection of this threshold is inherently subjective, as the outline of each pore cannot be defined with absolute accuracy. We selected the greyscale threshold of 100 for determining pore size because it minimized pore overlap (e.g., merging of two pores into one larger pore) while preserving what looks to be reasonable pore dimensions across the majority of the image area. While this image analysis approach cannot provide absolute accuracy, it can provide high reproducibility. The standard deviation of the ascertained average pore diameters from four different SEM images of each of the sensing layer and support layer PSi films was 2.7 nm and 1.7 nm, respectively. Since larger diameter pores are likely to be most important for the detection of proteins, we further determined that the pore size distribution in the sensing layer included a larger fraction of pores with diameter greater than 40 nm (56%) compared to the support layer (36%). We note that the fraction of larger pores can be increased if the applied current density used to form the PSi layer is increased. Table S1 summarizes the average pore diameter and thickness of each layer. Four individual PSi samples were analyzed to determine the pore size and thickness.

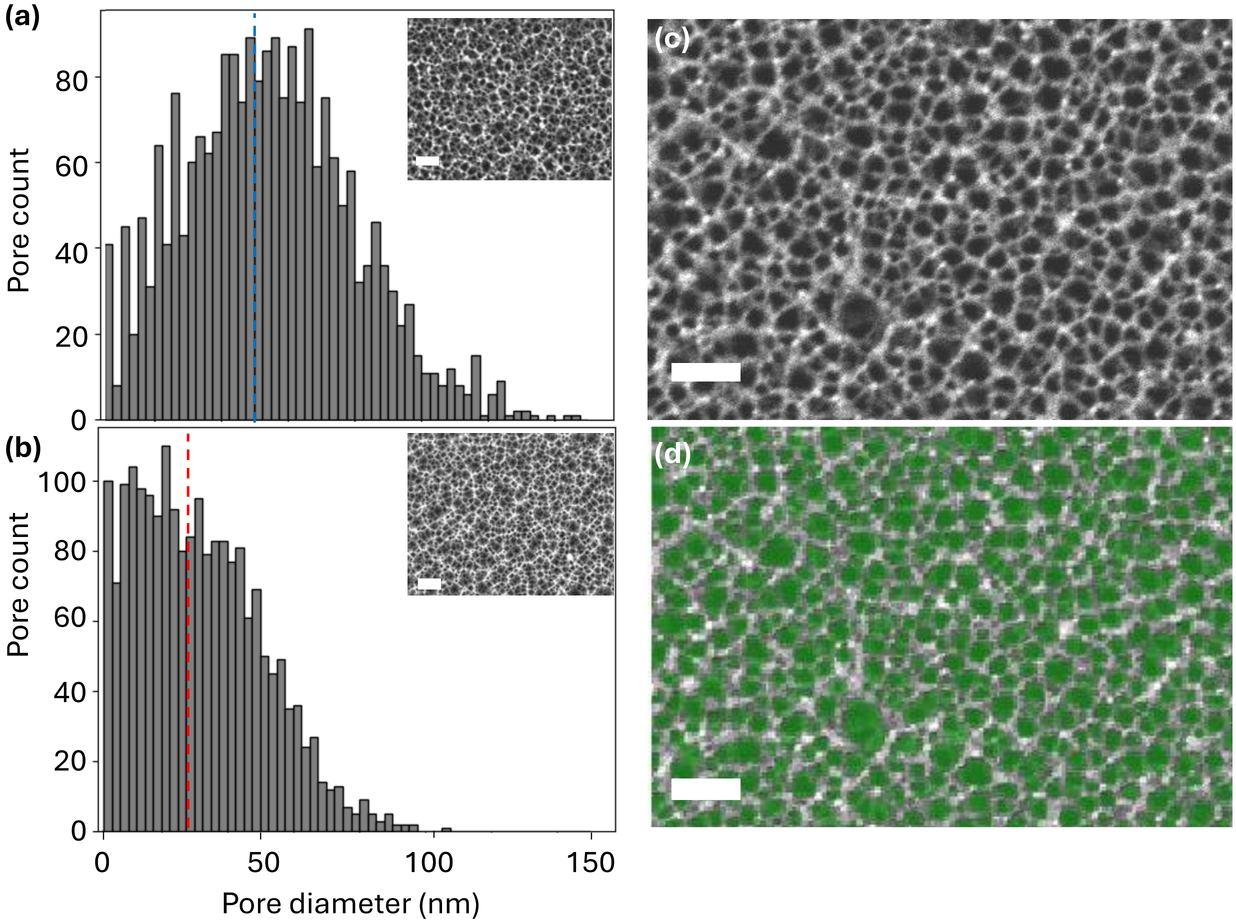

Figure S1. (a) Pore diameter distributions for PSi films formed with the same electrochemical etching conditions as the (a) top sensing layer (80mAcm^{-2}) and (b) middle support layer (40mAcm^{-2}) of the PSi FSM. The pore diameter distributions were determined based on image analysis of the SEM images shown in the insets of (a) and (b). The white scale bars in these inset images are 200 nm. The blue dashed line indicates the mean pore diameter of the top sensing layer and the red dashed line indicates the mean pore diameter of the middle support layer. (c) Enlarged top-view SEM image from the inset of (a). (d) Segmentation results of the SEM image in (c) using a greyscale threshold of 100; the regions defined as pores are shown in green.

Table S1. Average pore size, thickness, and fraction of pores larger than 40 nm for PSi films analyzed. Four individual PSi films were used to determine all parameters. Film thickness was determined from cross-sectional SEM image analysis with ImageJ, while all other parameters were determined from analysis of top view SEM images with MATLAB code analysis.

**Etching current density**	**80mAcm^{-2}**	**40mAcm^{-2}**
Mean pore diameter (nm)	47 nm	34 nm
Thickness (μm)	1.2 μm	25 μm
Fraction of pores > 40nm	56 %	36 %

2. Electropolished layer on bottom of PSi FSM

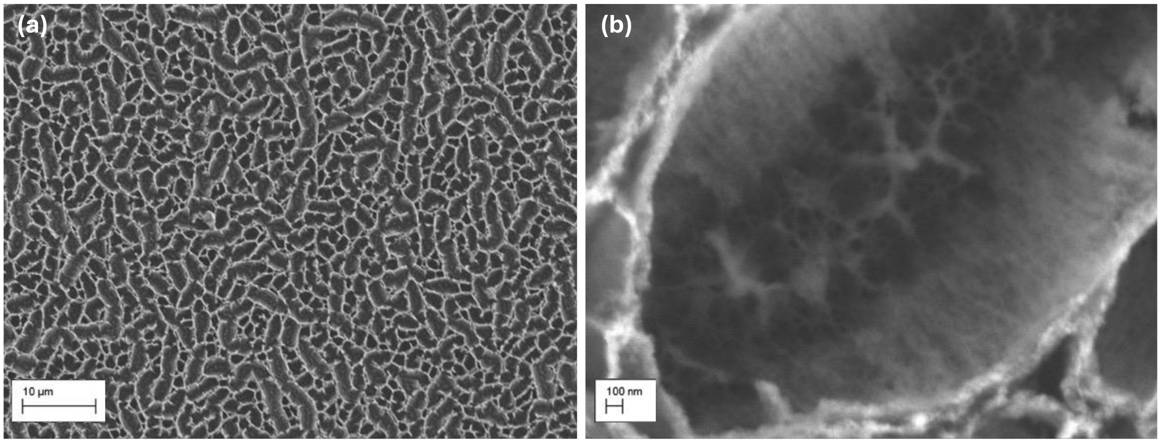

Figure S2. (a) SEM image of bottom of PSi FSM showing the large pores formed during the lift-off process that electropolishes the PSi and detaches it from the silicon substrate. (b) Zoomed in SEM image of representative pore in (a) in which the pores of the third layer of the PSi FSM are visible.

3. Reflectance spectrum and effective optical thickness of single-layer on-substrate PSi film

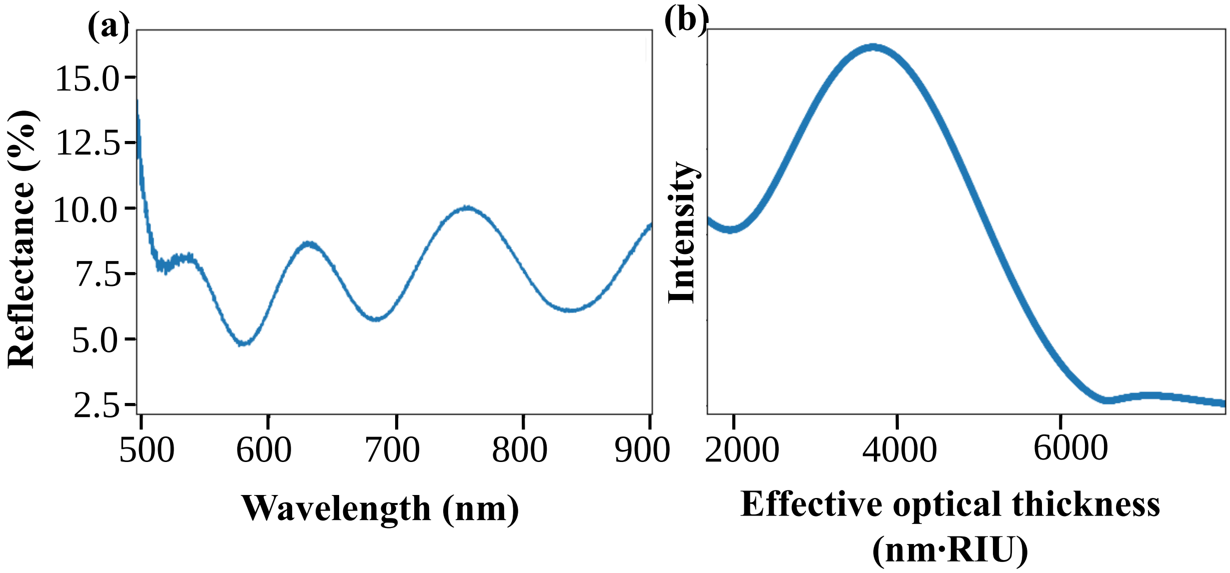

Figure S3. (a) Reflectance spectrum of single-layer on-substrate PSi film formed with the same electrochemical etching conditions as the top sensing layer of the PSi FSM. (b) Effective optical thickness (EOT) of the single layer on-substrate PSi film shown in (a). The single-layer on-substrate PSi film has the same EOT as the sensing layer of the PSi FSM.

4. Direct wetting of absorbent pad in PSi on paper sensor

To investigate the longer tail of the EOT decrease after a droplet of solution passes through the pores of the sensing layer of the PSi on paper sensor, we introduced 10 μL of water directly into the absorbent pad at the bottom of the PSi on paper sensor and monitored the real-time EOT changes. As shown in Figure S3, introducing water from the bottom of the PSi-on-paper sensor instead of the top leads to a very similar evolution of the EOT signal change. This suggests that the current sensor configuration and materials selection for the absorbent pad does not efficiently trap solutions and prevent their reabsorption into the PSi FSM above the absorbent pad. Hence, we believe that the currently measured drying time is not intrinsic to the PSi FSM and can therefore be significantly reduced with further improvements in the PSi on paper sensor design.

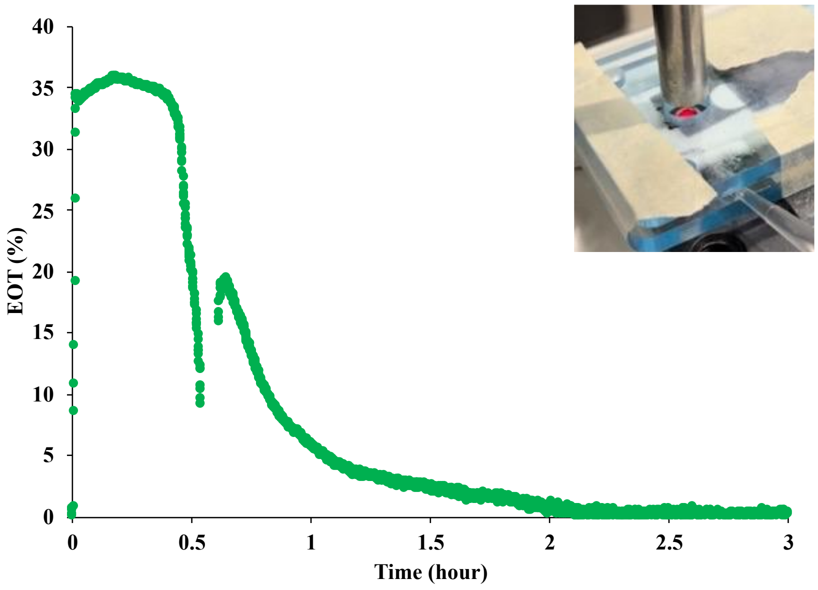

Figure S4. EOT change when water is added directly to the bottom absorbent pad of the sensor. Inset shows a picture of water being added to the absorbent pad with the PSi-on-paper sensor in the measurement system.

5. Non-specific binding

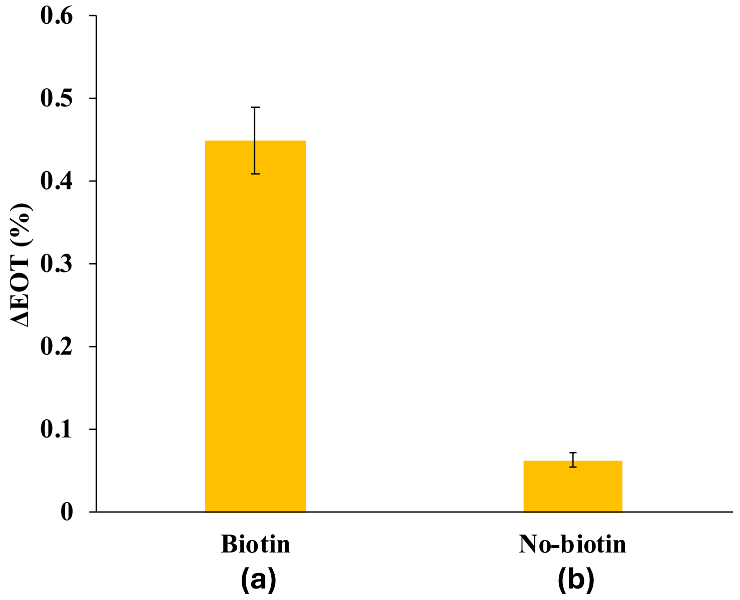

Figure S5. EOT change upon exposure of 10 μM streptavidin solution to PSi on paper sensors that were (a) functionalized with biotin as described in the main text and (b) functionalized with APTES but without biotin molecules. The larger EOT change for PSi on paper sensors functionalized with biotin demonstrates the important role of the capture agent. The small change in EOT for PSi on paper sensors that were not functionalized with biotin is most likely due to nonspecific binding.

6. Streptavidin sensing in on-substrate PSi films

In order to investigate how the magnitude of the EOT of biotin-modified PSi changes as a function of exposure time to a given concentration of streptavidin molecules, several on-substrate PSi films were fabricated with the same electrochemical etching conditions as the top sensing layer of the PSi FSM. These PSi films were then functionalized with biotin using the same procedure as described in the main text and subsequently exposed to streptavidin solutions with concentrations between 0 – 30 μM for times between 0 – 120 min. Adjusting the time duration of streptavidin exposure is straightforward in the PSi on substrate platform since it only requires incubating the sample for different lengths of time before rinsing away unbound species.

As shown in Figure S5, there is an approximately linear change in EOT as a function of time for each given streptavidin concentration exposed to the PSi on substrate sensors. We note that there is a decrease in EOT when 0 μM streptavidin solution is exposed to the sensor, which is due to corrosion of the PSi film in an aqueous solution, as discussed in other work[2]; other PSi surface passivation schemes can be used to mitigate this effect[3]. For the higher concentration streptavidin solutions, the increase in EOT with exposure time suggests that the PSi can accommodate the binding of more streptavidin molecules if given sufficient time. Hence, the future design improvements that will lead to longer contact (i.e., exposure) times for the PSi on paper sensor will lead to larger EOT changes when the sensor is exposed to a given concentration of molecules. Importantly, the time necessary to saturate the EOT change for a given target molecule concentration exposed to a PSi on paper sensor will be dramatically shorter than that necessary for the PSi on substrate sensor; prior work with a PSi membrane sensor in a different configuration suggested that optical signal changes saturate upon exposure to 5 μM streptavidin in 20 min. Hence,

we expect that future work will show larger EOT changes for the PSi on paper sensor without increasing the overall sensor response time[4] .

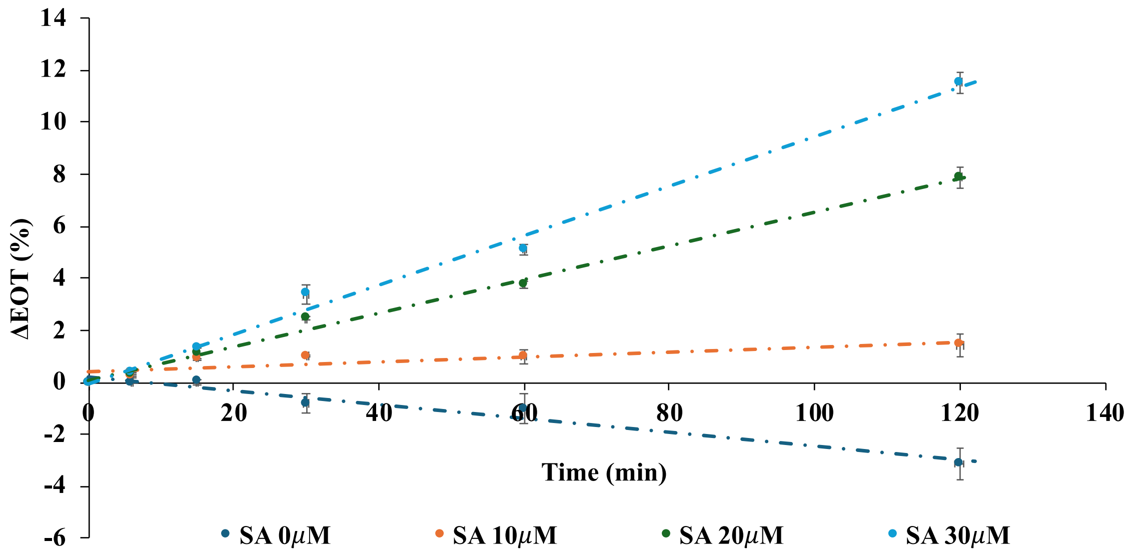

Figure S6. Biotin-modified PSi on silicon substrate films exposed to different concentrations of streptavidin (SA) solution for different exposure times. Linear fit lines are shown as a guide to the eye.

REFERENCES

(1) Ward, S. J.; Cao, T.; Zhou, X.; Chang, C.; Weiss, S. M. Protein Identification and Quantification Using Porous Silicon Arrays, Optical Measurements, and Machine Learning. *Biosensors (Basel)* **2023**, *13* (9), 879.

(2) Weiss Group Pore Size Distribution MATLAB Code. Available online: **https://my.vanderbilt.edu/vuphotonics/resources**.

(3) Sailor, M. J. Fundamentals of Porous Silicon Preparation: Preparation, Characterization and Application. In *Porous Silicon in Practice*; John Wiley & Sons, **2011**; pp 1–42. https://doi.org/10.1002/9783527641901.ch1.

(4) Layouni, R.; Choudhury, M. H.; Laibinis, P. E.; Weiss, S. M. Thermally Carbonized Porous Silicon for Robust Label-Free DNA Optical Sensing. *ACS Appl Bio Mater* **2020**, *3* (1), 622–627.

(5) Zhao, Y.; Gaur, G.; Mernaugh, R. L.; Laibinis, P. E.; Weiss, S. M. Comparative Kinetic Analysis of Closed-Ended and Open-Ended Porous Sensors. *Nanoscale Res Lett* **2016**, *11* (1), 395.
